# Preliminary engagement of a patient advisory board of African American community members with type 2 diabetes in a peer-led medication adherence intervention

**DOI:** 10.1186/s40900-020-00245-y

**Published:** 2021-01-06

**Authors:** Olayinka O. Shiyanbola, Betty L. Kaiser, Gay R. Thomas, Adati Tarfa

**Affiliations:** 1 Social and Administrative Sciences, University of Wisconsin-Madison School of Pharmacy, 777 Highland Avenue, Madison, WI 53705 USA; 2 Wisconsin Network for Research Support, University of Wisconsin-Madison School of Nursing, 701 Highland Avenue, Madison, WI 53705 USA

**Keywords:** Community engagement, African Americans, Diabetes, Medication adherence, Health equity

## Abstract

**Background:**

The Peers Supporting Health Literacy, Self-efficacy, Self-Advocacy, and Adherence (Peers LEAD) program is a culturally tailored educational-behavioral 8-week intervention that addressed psychosocial and sociocultural barriers to diabetes medication adherence in African Americans. A brief 3-week version of the Peers LEAD intervention used a community engagement approach to examine the feasibility and acceptability of the intervention amongst patient stakeholders.

**Main body:**

African Americans who were adherent to their diabetes medicines were paired with those who were non-adherent to their medicines. Together, they participated in the group and phone-based medication adherence intervention. Input from this brief intervention was important for the design of the remainder weeks of the 8-week program. The intervention targeted negative beliefs about diabetes, use of diabetes medicines, and offering culturally tailored peer support to improve medication adherence in African Americans. To receive input in the development and implementation of the program, we worked with community advisors and a peer ambassador board of African Americans who were adherent to their diabetes medicines. The peer ambassador board and community advisors reviewed intervention materials to ensure they were understandable and appropriate for the community. As well, they provided feedback on the process for intervention delivery.

**Conclusion:**

The active engagement of the peer ambassador board and community advisors led to a revised intervention process and materials for a medication adherence program for African Americans with type 2 diabetes.

## Plain language summary

Community engagement in the design of research interventions has long been advocated and supported by research agencies in the United States. Community engagement is even more important for minority populations whose voices are hardly sought in the development of public health programs. When minorities engage in decision making regarding program development, it increases the cultural relevance of the program to the community and is an important strategy for increasing health equity. In this paper, we describe the process of obtaining the input of African Americans with type 2 diabetes who were successfully taking medicines in the design of a program for African Americans with type 2 diabetes who had challenges taking medicines. In this brief 3- week program for individuals with diabetes, we targeted an increase in the ability to understand diabetes health information. As well, participants learned skills on developing confidence in taking medicines and talking to health providers about diabetes and medicines. Over six months, we worked together with a peer ambassador board and community advisors to ensure that our program materials and intervention processes were appropriate for the African American community. Through this approach, we were able to make changes to the 8- week program including adding a physician-led education session; and phone call sessions to address salient sociocultural barriers to nonadherence among African Americans, including provider mistrust and talking to family and friends. As well, we changed the program logo and redesigned the group education sessions to have basic information about diabetes before discussing medicines. The expected impact of the 8- week program is to improve medication adherence and diabetes outcomes for African Americans. Our goal is to continue to learn from our partnership with minorities whose voices are important in the design of culturally relevant public health programs.

## Background

Community engagement in research, including inclusion in the process of developing and implementing an intervention, is increasingly being recognized and supported by the National Institute of Health, as well as an independent, non-profit, non-government US organization authorized by the 2010 Patient Protection and Affordable Act, the Patient-Centered Outcomes Research Institute [1–3]. Community engagement during a research process increases the relevance of the study to the intended participants and enhances the quality of the research [4]. Studies have shown that including patients and members of a community as partnering stakeholders in research can contribute to the identification of pertinent research questions that might have eluded the investigator; can help design recruitment, retention, and research protocols; can improve the effectiveness and efficacy of health promotion interventions, and can enhance the dissemination of study results to the community [5, 6].

Studies have described community engagement as an important strategy for understanding and reducing health disparities among underserved populations [7, 8]. Type 2 diabetes is one of the most serious health conditions disproportionately affecting African Americans. Compared with the general US population, African Americans are more affected by type 2 diabetes especially when compared to non-Hispanic whites. For example, African Americans are twice more likely to be diagnosed and two to four times more likely to suffer from diabetes complications such as blindness, kidney disease and lower limb amputations [9–12]. One of the most important means of managing type 2 diabetes is taking medicines.

Medication adherence is defined as patients taking their medications as recommended by their health care provider(s) [13]; a disease self-management behavior critical for managing a chronic illness like type 2 diabetes. African Americans have lower rates of medication adherence compared to non-Hispanic Whites [14] which is a prominent contributor to diabetes disparities [15]. Current medication adherence interventions may not work well for African Americans with type 2 diabetes for several reasons including a lack of culturally specific content and tailoring of interventions [16], access and socioeconomic barriers, distrust of the healthcare system, etc. [17] Engaging African Americans in the development and implementation of a medication adherence intervention has the potential to improve the cultural relevance and effectiveness of the intervention, and subsequently, reduce prevalent health disparities within the community. As well, there is the potential benefit of greater community satisfaction and ownership and investment in the intervention.

Various methods are used to involve members of communities in health improvement initiatives and it is useful for studies to provide descriptions of the approaches and methods employed [18]. By reporting the initiatives used in studies, other researchers, policymakers, and health programs can replicate the interventions, as well as evaluate the impact of the program. Therefore, the objective of this paper is to describe the process of engaging with African American community stakeholders and community advisors in the development and refinement of a culturally tailored intervention to provide African Americans with diabetes information, skill-development, and motivation to address psychosocial/sociocultural concerns and enhance diabetes medication adherence [19]. We describe the community-academic engagement process for the development and implementation of the Peers Supporting Health Literacy, Self-efficacy, Self-Advocacy, and Adherence intervention. The first 3 weeks of the 8-week program was conducted to test for the feasibility and acceptability of the program.

## Main text

### Design of the Intervention

#### The intervention

Peers Supporting Health Literacy, Self-efficacy, Self-Advocacy, and Adherence (Peers LEAD).

The Peers LEAD program is a culturally tailored educational-behavioral 8-week intervention that addresses psychosocial/sociocultural barriers to diabetes medication adherence. In this study, we designed and implemented the first 3 weeks of the intervention. This brief intervention entailed three sessions which included two group education sessions (Session 1 and 2) and one phone call between peer buddies and peer ambassadors (Session 3). The first 2-h group session (Session 1) was facilitated by an African American dietician/diabetes educator. The second session was led by an African American pharmacist. The goal of the group sessions was to discuss illness beliefs with a focus on the cause and consequence of diabetes, including sociocultural influences that led to these beliefs. The pharmacist education session specifically focused on beliefs regarding taking diabetes medicines, reasons for nonadherence, and communicating with a pharmacist or other healthcare provider about diabetes medicines. Finally, peer ambassadors called peer buddies for a 15–30-min phone call to discuss ways to cope with diabetes and how to ask questions from healthcare providers. Peer ambassadors were men and women 30–65 years old with type 2 diabetes who self-identified as Black/African American, could speak and read English, self-reported use of prescribed oral diabetes medication, and were adherent to taking these medicines. Peer buddies were also 30–65 years old men and women with type 2 diabetes who self-identified as Black/African American, could speak and read English, self-reported use of prescribed oral diabetes medication, but were not adherent to taking these medicines.

The stakeholder groups involved in the design of the study, the delivery of the study, and collecting feedback from community members included the Community Advisors for Research Design and Strategies (CARDS), who are part of the Wisconsin Network for Research Support (WINRS), and the peer ambassador board which consisted of African Americans with type 2 diabetes who were taking their medicines. The inclusion criteria for the peer ambassador board included self-identifying as African American or Black, being between the ages of 30 to 64 years old, taking oral diabetes medicines, communicating in English, and being diagnosed with type 2 diabetes for at least one year.

The main outcome of the study was to improve medication adherence among African Americans who had challenges with medication adherence using a peer-led intervention. Therefore, as part of the intervention, African Americans included were either a peer ambassador or peer buddy. Peer ambassadors are described as African Americans who were adherent to taking their oral diabetes medicines. Peer ambassadors were considered adherent if they scored 25 out of 25 points on the Medication Adherence Report (MARS-5 scale) [20]. African Americans with lower than 25 points on the MARS-5 scale, which means nonadherent to their oral diabetes medicines were grouped as peer buddies. The plan of the study was to test the feasibility of the program among 10 peer ambassadors and 10 peer buddies paired based on gender and age, information collected during the participant screening process. The interventions were peer-led, therefore the peer ambassadors provided support to peer buddies. Peer ambassadors were matched to buddies based on age ± 15 years old, and gender. During the recruitment of the peer ambassador, the program assistant informally assessed the peer ambassador’s personality, communication, and interpersonal skills during initial discussions. Hence, the peer ambassadors acted as a peer leader and support individual, and were trained to actively support peer buddies self-advocacy in patient-provider relationships, share their experiences managing diabetes, provide social support, and enhance self-efficacy. Recruitment and retention information of the peer ambassadors and peer buddies are included elsewhere [21]. The peer ambassadors had a dual role in this study by being part of the peer ambassador board and provided feedback on the design and implementation of the intervention, as well as provided peer support to peer buddies having challenges taking their prescribed medicines.

Table 1 shows the plan for weekly interventions that the peer ambassadors and peer buddies participated in either as group education or as a one-on-one peer phone-based session. For the 3-week program, since the intervention consisted of structured group diabetes education and follow-up peer support with a peer ambassador, the peer ambassador attended each group education session with their peer buddies, to learn together, and build social connections. As well, peer ambassadors completed one follow-up phone call with their peer buddy. Using surveys and interviews, the research team (including an outreach specialist, research assistant, and the PI), evaluated feedback from each group education session, and their overall perception of the program. Details of the intervention results and evaluation are presented elsewhere [21].

**Table 1:** The Peers Supporting Health Literacy, Self-efficacy, Self-Advocacy, and Adherence (Peers LEAD) program 8-week intervention

Week	Intervention	Details of intervention delivery
**Week 1**	**Discuss patient self-management goals and intervention details. Target negative illness beliefs with a focus on the cause and consequence of diabetes.**	**A 2-h in-person group session with peer ambassador and peer buddy led by an African American diabetes educator**
**Week 2**	**Reframe medication beliefs to decrease medication concerns and increase the necessity of medicines. Address reasons for nonadherence.**	**A 2-h in-person group session with peer ambassador and peer buddy led by an African American pharmacist**
**Week 3**	**Discuss self-efficacy, positive living, and coping with diabetes.**	**A one-on-one phone call between paired peer ambassador and peer buddy**
Week 4	Provide support for addressing fear, frustration, and emotional distress.	A one-on-one phone call between paired peer ambassador and peer buddy
Week 5	Discuss self-advocacy in provider communication/relationship building.	A one-on-one phone call between paired peer ambassador and peer buddy
Week 6	Discuss family/community bonding and maintaining cultural experiences.	A one-on-one phone call between paired peer ambassador and peer buddy
Week 7	Discuss hemoglobin A1C as well as blood sugar, diet, and exercise.	A one-on-one phone call between paired peer ambassador and peer buddy
Week 8	Re-examine patient goals.	A one-on-one phone call between paired peer ambassador and peer buddy

The bolded data show emphasis of the 3-week intervention conducted in this study

#### Intervention conceptual approach

The conceptual approach that guided the design of the intervention was the extended self-regulatory model [22] and the information-motivation-behavioral skills model [23]. The self-regulatory model addresses beliefs about medicines and illness and integrates with the information-belief component of the latter model. This intervention addresses misinformation on diabetes and medicines among African Americans [22, 24]. The information-motivation-behavioral skills model focuses on constructs needed for successful diabetes adherence [25] including *information* “an initial prerequisite for enacting a health behavior”, *motivation* that takes into account beliefs about the intervention outcome and attitudes toward adherence [23, 26] as well as perceived social support for engaging in a behavior [27], and *behavioral skills* which emphasize increasing self-efficacy [23]. The intervention process for the initial 3 weeks of the Peers LEAD program was grounded within these theoretical frameworks.

The stakeholders involved in the development and implementation of the intervention included the academic collaborators and peer ambassador board (development) and the Community Advisors for Research Design and Strategies - Wisconsin Network for Research Support and peer ambassador board (implementation and refinement). We discuss the role of each stakeholder in the intervention process.

#### Intervention development

##### Academic collaborators (research team)

An interdisciplinary team of individuals with expertise in medication adherence and chronic illness self-management among African Americans served as experts and academic collaborators for the program. The primary investigator had expertise in illness perceptions, beliefs about medicines, and medication adherence among underserved populations and guided the development of the intervention. The academic collaborators brought expertise and experience in various aspects including designing and implementing African American community-based research interventions, addressing cultural health beliefs, and research focused on contextual challenges that make it difficult for underserved populations to manage diabetes.

The PI and academic collaborators met at least once a month for 6–8 months to discuss the development of the manuals, guides, and materials for the intervention; how to engage with the community on the intervention implementation; and the process for incorporating the African American’s community’s perspective in the development and implementation of the intervention.

##### Peer ambassador board

The Peers LEAD intervention was designed using a community engagement approach. This design has been used in our prior work to identify critical issues related to medication adherence and diabetes self-management among African Americans with type 2 diabetes, which informed the development of the intervention [19, 28, 29]. The use of group education sessions was informed by our prior discussions with African American community members with diabetes [28]. The community engagement approach allowed us to address patient-related psychosocial and sociocultural barriers related to medication adherence [28, 29]. The peer ambassador board comprised of nine African Americans with type 2 diabetes who were engaged with the research team in an advisory role, as well as part of the team delivering the intervention in their role as peer ambassadors. The responsibilities of the peer ambassador board included providing community feedback on the intervention content and process. Four 2-h stakeholder meetings (one orientation, one training meeting, and two feedback meetings) were led by the PI. Meetings were held at convenient times using a local community center. To compensate for their time and help with transportation costs, peer ambassadors were paid $25 per hour for each meeting they attended.

The inclusion criteria for being on the peer ambassador board (same as been a peer ambassador) included (1) Men and women 30–65 years old with type 2 diabetes who self-identified as Black/African American and could speak and read English. (2) Participants who self-reported use of prescribed oral diabetes medication and were adherent to these medicines. Using purposive sampling, we worked with our community partners to recruit peer ambassadors from the community. Flyers and word of mouth through a community partner, the African American cultural diversity program of a senior center was the major recruitment method. Peer ambassadors consented to be part of the program through a signed investigator responsibility form approved by the University’s Institutional Review Board. Peer buddies consented to their participation through a written informed consent form.

### Evaluation and refinement of the intervention

We outline the intervention implementation processes that the peer ambassadors were involved, including an ethics training, an orientation to the program, and training to prepare them for their role as ambassadors. We collaborated with the Wisconsin Network for Research Support in facilitating these processes.

The Wisconsin Network for Research Support (WINRS) is a patient and community engagement resource based at the PI’s institution School of Nursing [30], who consulted with our research team on all peer ambassador board meetings. WINRS staff developed and co-facilitated the board orientation and training meetings. Since 2010, WINRS staff has developed and delivered more than 20 tailored orientations for lay advisory groups [31]. In addition, they have planned and facilitated over 250 meetings between researchers and lay advisory boards. The WINRS staff helped design the logistics of the program, developed agendas and activities for peer ambassador meetings, wrote facilitator scripts/guides for each stakeholder meeting and debriefed with the research team throughout the program to problem-solve emerging issues, as well as identify strategies for improving the program processes and materials. WINRS staff helped with the revision of the intervention materials based on feedback from peer ambassadors. They also facilitated one meeting between the research team and the Community Advisors on Research Design and Strategies (CARDS)®^.^ The CARDS® is part of WINRS and includes about 8–10 community members recruited from programs at local community centers, such as women’s groups and food pantries. They provide unique feedback on a wide variety of project materials, including recruitment materials and strategies, survey and focus group questions, intervention design, and content and usability of websites and mobile apps. These individuals may or may not have diabetes, or be African American, which differentiates them from the peer ambassador board. The CARDS® provided feedback on the recruitment materials for peer ambassadors and peer buddies, including the flyers and peer ambassador job description (Table 2).

**Table 2:** Feedback from Community Advisors on Research Design and Strategies (CARDS) ^a^

Topic	Comments/Suggestions	How the research team adapted the suggestions
**Peer buddy recruitment flyer**	The title does not grab attention; CARDS noted they did not immediately understand participation was needed in the study	Clearly state that this is a research opportunity specifically for African Americans
	Excessive wording	Trimmed down wording
	Clarify that participants can be on insulin	Re-specified the inclusion criteria to include those on oral medicines or insulin
	How many sessions and how long is each? How many total hours and weeks will this project require?	Included more detailed information about the sessions
	Emphasize that the first two sessions are educational– may be appealing to some	Renamed the pharmacist and dietician sessions to include the word “education”
**Pay policy for peer ambassadors**	Clearly specify pay policy if peer ambassadors miss study activities or arrive late/leave early	Included information about maximum earning amount and absence/late arrival/early leaving policy
**Peer ambassador job description**	What is “ethics training”? Several CARDS expressed confusion (“Teaching me how to be an ethical person? An ethical African American?”)	Clarified ethics training description and why it was a requirement for the peer ambassador
**Ethics training for peer ambassadors**	Can it be called a “certification”? (“I get training and a certificate that I could use elsewhere.”)	Created a certificate and provided it to those who completed the ethics training
**Title of peer participants**	Some CARDS members preferred the title ‘peer buddy’. Some CARD members felt that “peer ambassador” was formal and off-putting, but other community advisors thought it sounded very “official” and liked it.	Retained “peer ambassador” name as advised. Peer buddy was retained for use in describing participants with medication adherence challenges.
**Program location**	Several CARDS members were concerned about location, especially if sessions were at rush hour	Confirmed with peer ambassadors that location and timing of meetings were convenient.

^a^CARDS provided feedback on project items including the recruitment flyer and peer ambassador job description to the research team at a community education center on December 11, 2017

#### Peer ambassador’s intervention involvement

##### Human subjects training of peer ambassadors

To be able to participate in the implementation of the intervention, the peer ambassadors completed a 2-h community-based research ethics training to meet the PI’s institution (a large public University in Midwest USA) regulation for their role as a peer ambassador. The training was delivered by the PI or research specialist using an adaptation of an ethics research training manual [32]. This training covered human subjects research rules and regulations, history of research abuses, ethical principles, regulations, and institutional review boards, research with communities, and the informed consent process, with a specific focus on protecting subjects’ information, privacy, and confidentiality. All peer ambassadors received a certificate to indicate their completion of the research ethics certification. At the end of the training, peer ambassadors also read their job descriptions, which they had already known about informally during screening. The PI was available to answer questions about their role and responsibilities, after which the peer ambassadors signed a membership agreement to indicate their participation.

##### Orientation of peer ambassadors to the program

A week after their research ethics training, peer ambassadors completed an interactive 2-h orientation that described the project goal, outlined the roles of all project stakeholders, and provided opportunities for them to practice their roles. A role-play activity led by the PI and a member of the research team demonstrated the peer ambassador role in listening and supporting a peer buddy. After discussing the simulated role play, peer ambassadors then conducted their own role plays amongst themselves using brief scripts that modeled a peer ambassador - peer buddy interaction. At the end of the meeting, the peer ambassador membership agreement was reviewed again to answer questions about the position.

##### Peer ambassador training

Peer ambassadors completed one additional training session to prepare them for their phone call with peer buddies. Role-playing activities were the main mechanism for this training. They worked in pairs, with one person initially playing the role of peer ambassador and the other person playing the role of peer buddy. Using a telephone call guide that we developed for this program, each person in the peer ambassador role practiced a 7 to 8-min conversation with a peer buddy. The roles were then switched so that everyone could experience using the telephone call guide in a conversation with a potential peer buddy. Midway through the role-play and afterward, we solicited detailed feedback on the activity telephone guide. We also asked peer ambassadors to reflect on their role and discuss the easiest and hardest things about talking with a potential peer buddy on the phone. We used peer ambassador feedback and comments to revise the telephone guide.

## Results

### Intervention refinement

#### Peer ambassador board feedback on intervention

**Fig. 1:**
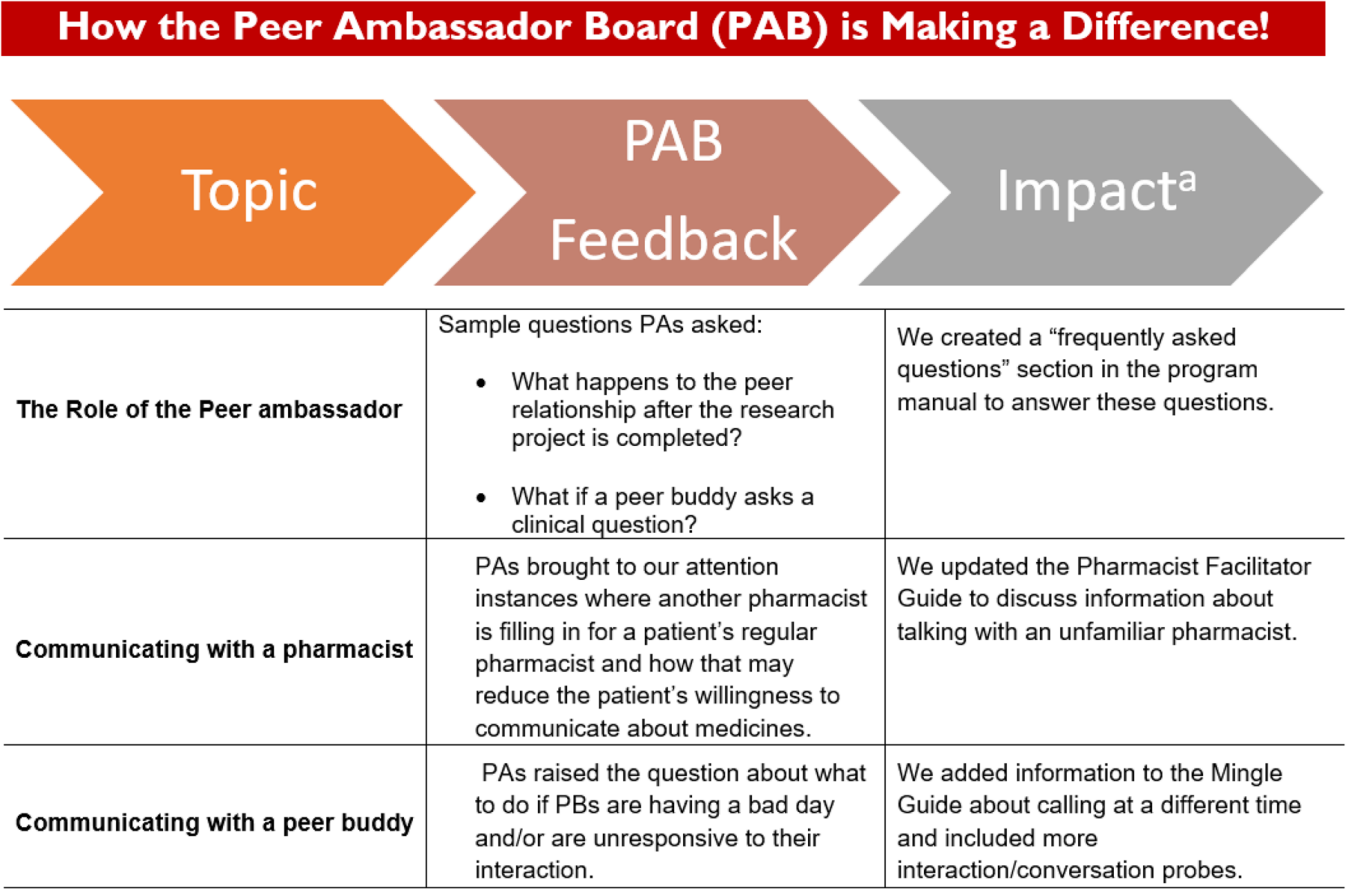
A Sample of the Incorporation of Feedback from The Peer Advisory Board Meetings. ^a^Impact - how the researchers responded to the peer ambassador board feedback

After the orientation and training meetings, our subsequent meetings with peer ambassadors focused on getting feedback on the group education sessions and phone calls. Specifically, we discussed session content (which session information was useful, whether the information was interesting, and what was missing from the session and needed to be added) and format (length of the session and mix of discussion, lecture, and question time). Other discussion items included, what worked well and could have been better during the phone call with their peer buddy, the process the research team used in pairing peer ambassadors and buddies, and the possible addition of an orientation for peer buddies. Some of the changes made based on the feedback included adding a physician-led education session to the intervention; using the phone call sessions to address more salient sociocultural barriers to nonadherence among African Americans, including provider mistrust and talking to family and friends. As well, we changed the program logo, redesigned the group education sessions to have basic information about diabetes be discussed first before discussing diabetes medicines, added an orientation to the program for peer buddies as well, ensured that there was a frequently asked question in the peer ambassadors program manual, and included a guide for the peer ambassador to interact, communicate and build rapport with a peer buddy for the first time. Finally, we included information that would guide participants on how to communicate with a pharmacist they were unfamiliar with. After we made changes based on the feedback, we highlighted how we were using the advice by providing a handout called *“How the Peer Ambassadors Are Making a Difference*”, based on a template provided by WINRS (Fig. 1). The handout was a simple table with 2–3 examples of topics we discussed at a previous meeting, a summary of group feedback, and how the research team acted on the feedback. The final meeting wrapped up with the research team helping peer ambassadors include a description of their membership on the peer ambassador board as part of a resume, LinkedIn profile, or job application; the resume template was provided by WINRS.

Peer ambassadors also had comments about their experience acting in the role. There were verbatim comments, “*You got to learn some things about diabetes that you didn’t know. And you get to get with a peer buddy, and they give you information as well. You feed off of each other. The information that you get, you receive as well as you give*”

“*And like she said, you get to meet somebody. You get to meet a lot of peoples. I got to meet some peoples that I didn't know up in here. Like I say, it was a great experience, and it's something that if you have nothing to do, and you have diabetes, and you figure that you want to learn more about diabetes, this is, this could be a good experience for you”*

The strengths of this study are the engagement of African Americans with a chronic disease (as peers and in an advisory role) in the development, implementation, and refinement of an intervention to address medication adherence among African Americans having challenges; as well as involving community advisors on research strategies in enhancing intervention materials. There are some limitations in this study. First, there was no formal process to assessing the communication and interpersonal skills of peer ambassadors. Second, this study only considers the perspectives of community advisors in the community, as well as African American individuals with type 2 diabetes. The members of the CARDS may not reflect the actual community of interest as though most of them identified as Black/African American, they may not have been diagnosed with type 2 diabetes and/or be taking prescribed diabetes medicines. While their input was important as community advisors trained to offer feedback on research materials, it is limited compared to the peer ambassador board who were diagnosed with type 2 diabetes, taking diabetes medicines, and identified as Black/African American. We did not consider the perspectives of other several important stakeholders in patient-centered diabetes medication adherence interventions, including community organizations, diabetes educators, and care partners. We used a self-report measure to estimate medication adherence, which may lead to overestimation of nonadherence. There is limited information regarding how the community engagement was sustained over the course of this feasibility study. Finally, we did not evaluate the peer ambassador’s perspective of their engagement and partnership throughout the study. This is now considered in our current work.

## Conclusions

This paper discusses how academic collaborators, community advisors on research strategies, and African American community members informed the design of a peer-led medication adherence program, by providing feedback at varying stages of the intervention. The paper describes the initial engagement process of a peer ambassador board consisting of African American community members with type 2 diabetes who served as members of a board in an advisory role, as well as a role of a peer ambassador providing peer support to a buddy. Based on feedback received, we were able to make additions and changes to the subsequent full 8- week program including adding a physician-led education session; using the phone call sessions to address salient sociocultural barriers to nonadherence among African Americans, including provider mistrust and talking to family and friends. As well, we refined the program logo, redesigned the group education sessions to allow the physician-led session occur first chronologically before the pharmacist and diabetes educator session, and specify discussion on basic information about diabetes in the physician-led session. By including community members at each stage of the study, we tailored the program to the specific needs of African Americans living with type 2 diabetes in the community. The next step in this community engagement process is our current study to further engage the Peer Ambassador Board in the implementation of a full 8-week intervention to address culturally informed beliefs, self-efficacy, health literacy and medication adherence with a new cohort of peer buddies. Future studies should consider community engagement in intervention processes, to help mitigate other social, economic and access issues that may make medication adherence interventions not work for African Americans.

## Data Availability

Data sharing is not applicable to this article as no datasets were generated or analyzed during the current study.

## Abbreviations

Peers LEAD: Peers Supporting Health Literacy, Self-Efficacy, Self-Advocacy and Adherence
CARDS: Community Advisors on Research Design and Strategies
WINRS: Wisconsin Network for Research Support
